# Treatment of phantom limb pain (PLP) based on augmented reality and gaming controlled by myoelectric pattern recognition: a case study of a chronic PLP patient

**DOI:** 10.3389/fnins.2014.00024

**Published:** 2014-02-25

**Authors:** Max Ortiz-Catalan, Nichlas Sander, Morten B. Kristoffersen, Bo Håkansson, Rickard Brånemark

**Affiliations:** ^1^Biomedical Engineering Division, Department of Signals and Systems, Chalmers University of TechnologyGothenburg, Sweden; ^2^Department of Orthopaedics, Centre of Orthopaedic Osseointegration, Sahlgrenska University HospitalGothenburg, Sweden

**Keywords:** phantom limb pain, augmented reality, virtual reality, myoelectric control, electromyography, pattern recognition, neurorehabilitation

## Abstract

A variety of treatments have been historically used to alleviate phantom limb pain (PLP) with varying efficacy. Recently, virtual reality (VR) has been employed as a more sophisticated mirror therapy. Despite the advantages of VR over a conventional mirror, this approach has retained the use of the contralateral limb and is therefore restricted to unilateral amputees. Moreover, this strategy disregards the actual effort made by the patient to produce phantom motions. In this work, we investigate a treatment in which the virtual limb responds directly to myoelectric activity at the stump, while the illusion of a restored limb is enhanced through augmented reality (AR). Further, phantom motions are facilitated and encouraged through gaming. The proposed set of technologies was administered to a chronic PLP patient who has shown resistance to a variety of treatments (including mirror therapy) for 48 years. Individual and simultaneous phantom movements were predicted using myoelectric pattern recognition and were then used as input for VR and AR environments, as well as for a racing game. The sustained level of pain reported by the patient was gradually reduced to complete pain-free periods. The phantom posture initially reported as a strongly closed fist was gradually relaxed, interestingly resembling the neutral posture displayed by the virtual limb. The patient acquired the ability to freely move his phantom limb, and a telescopic effect was observed where the position of the phantom hand was restored to the anatomically correct distance. More importantly, the effect of the interventions was positively and noticeably perceived by the patient and his relatives. Despite the limitation of a single case study, the successful results of the proposed system in a patient for whom other medical and non-medical treatments have been ineffective justifies and motivates further investigation in a wider study.

## Background

Phantom limb pain (PLP) is a common and deteriorating condition suffered by ~70% of amputees (Dijkstra et al., 2002), and regardless the cause of amputation (Clark et al., 2013). In recent years, virtual reality (VR) has been used to treat PLP as a more technologically sophisticated version of the well-known “mirror” therapy introduced in 1996 (Ramachandra and Rogers-Ramachandra, 1996). VR has clear advantages over the physical constraints imposed by the conventional mirror box, as it allows a wider range of motion and rehabilitation exercises. In addition, VR allows interactive games that challenge patients with varying levels of difficulty, while keeping them entertained and motivated (Sveistrup, 2004). Contemporary reviews of the use of VR in neuromuscular rehabilitation are given in Sveistrup (2004), and Holden (2005).

To date, VR mirror therapy has relied on patients commanding the same motor execution in both limbs. A virtual representation of the missing limb is then created to match the motions of the contralateral limb, thus delivering visual feedback (Murray et al., 2006a,b; Mercier and Sirigu, 2009; Bach et al., 2010). Since the sound limb is required, this approach is only suitable for unilateral amputees. The patients have no direct volitional control of their phantom limb virtual representation. Instead, they simultaneously execute the same motions in both limbs. In this setup, the real effort and commitment of the patient to produce phantom limb motions is not part of the intervention, i.e., the mirror limb will move as long as the sound limb does, and regardless of the intention of the phantom limb. Additionally, it has been suggested that the variable efficacy of this therapy across subjects is mainly due to the difference in individual susceptibility to the visual feedback, rather than the physiological condition itself (Mercier and Sirigu, 2009). We hypothesize that the higher degree of realism provided by augmented reality (AR), together with direct volitional control through the prediction of motion intent using myoelectric signals at the stump, could improve the efficacy of this therapy. Furthermore, the addition of game control by phantom limb motions should help to engage the patient in executing these movements and, since only the amputated limb is involved, it is also suitable for bilateral amputees.

VR-based treatment in which the virtual limb is controlled by the affected side has been previously explored with motion tracking technology (Cole et al., 2009), which inherently, and considerably, restricts the amount of predictable motions. Here we show that myoelectric pattern recognition allows for the accurate prediction of hand, wrist, and elbow motions as intended in an intact limb.

The utilization of the stump musculature to control conventional myoelectric prostheses has been long thought to reduced PLP (Lotze et al., 1999), despite that most commonly, the controlling muscle contractions are not originally related to the end actuation (i.e., in a trans-humeral amputee, an electrode over the biceps muscles controls the closing of a prosthetic hand). However, even if the musculature for physiologically appropriate actuation is no longer present, it has been shown that amputees are able to distinguish between imagining a phantom movement, and actually executing it. This suggests that the ability to naturally execute a movement is maintained after amputation, but more importantly, the effect on neuroplasticity and inter-hemispheric communication is different when practicing motor execution and motor imaginary (Raffin et al., 2012a,b). Experiments with implanted neural interfaces, which rely on the physiology of motor execution, have been shown to reduce PLP (Di Pino et al., 2012). This supports the use of direct volitional control through myoelectric signals at the stump, with the advantage that the system presented here is non-invasive, and allows the equivalent to a physiologically appropriate control (i.e., muscle synergies generated with the intention of closing the missing hand, results in closing of the virtual hand).

It has been suggested that incongruencies in the visual stimulus and sensory perception produce varying results in terms of pain relief, in some cases increasing it (Desmond et al., 2006). This problem is avoided in our proposed myoelectrically controlled AR environment (MCARE), where a conventional webcam captures the whole environment around the patient and integrates it in the rehabilitation task. To the best of our knowledge, this is the first time that AR, gaming, and the prediction of motion intent using myoelectric pattern recognition have been used together as a treatment for PLP. Comprehensive reviews of PLP are given in Nikolajsen and Jensen (2001), and Flor et al. (2006). In this work, the results of using MCARE in a chronic, treatment-resistant PLP patient are reported.

A chronic PLP patient for whom other treatments have proven ineffective was recruited to this study. The patient (male, 72 years old) lost his arm just below the elbow joint in 1965 due to a traumatic injury. He has experienced PLP since the amputation and reported a strongly closed fist as the permanent posture of his phantom hand. The PLP has continued over the years, despite conventional mirror therapy, different drug-based treatments, acupuncture, and self-suggested hypnosis. The patient has reported living with constant burning pain of an intensity of 3 on a scale from 0 to 10 (SF-MGPQ; Melzack, 1987), with episodes that escalated up to the maximum intensity approximately every hour for a few minutes, reported as excruciating pain. In addition, the patient was normally woken at night due to intense episodes of pain.

## Methods

### Pain tracking

Pain perception was monitored after every session using the short-form McGill pain questionnaire (SF-MGPQ) (Melzack, 1987) translated into Swedish (Burckhardt and Bjelle, 1994). The questionnaire was administered by a facilitator, with the exception of pain intensity where the patient noted the rating directly on the visual analogue scale. A percentage of total time at each level of pain was also reported. Additionally, the patient was free to self-report any comments on the system and the treatment.

### Control source

The prediction of motion intent was made using BioPatRec, an open source platform initially developed for advanced prosthetic control strategies based on pattern recognition algorithms (Ortiz-Catalan et al., 2013). The myoelectric activity at the patient's stump was utilized as the sole input to determine the intended phantom limb motions. Once the aimed motion is known, this can be used to command a variety of virtual environments and robotic devices. A custom-made AR environment was developed for this study to interface with BioPatRec and allow the patient to visualize himself (in real-time) with a virtual arm superimposed on his stump. The AR environment uses a conventional webcam which inputs a video feed that is analyzed to track a fiducial marker, thus allowing the virtual arm to remain in the anatomically correct position while the patient moves (see video in Additional File 1). The fiducial marker can be printed with a conventional printer. The virtual arm is superimposed on the marker and changes scale and rotation based on the tracking of the marker. These parameters can be also adjusted in real-time with the keyboard in order to improve the fitting of the virtual arm.

### Myoelectric recordings

**Figure 1:**
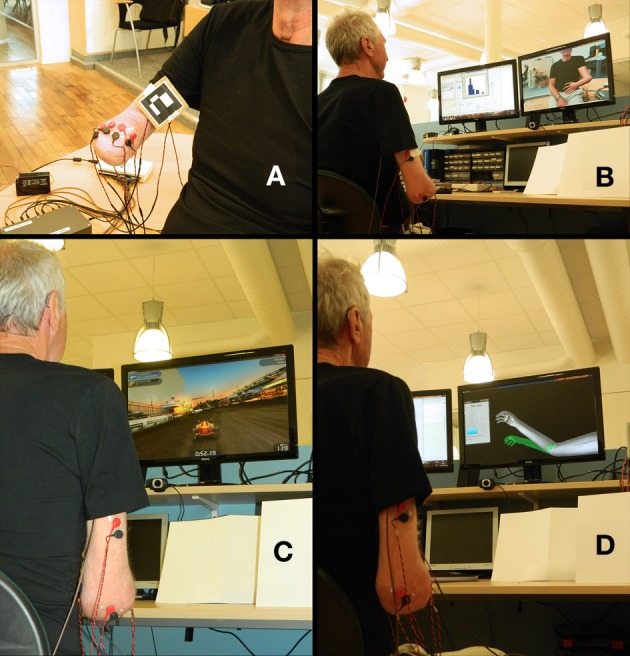
**Setup for the myoelectrically controlled augmented reality environment (MCARE). (A)** Surface electrodes and a fiduciary marker placed at the stump. **(B)** Environment captured by the webcam and displayed on a computer screen, with the addition of the virtual limb superimposed on the fiduciary marker. **(C)** Patient playing a racing game in which he drives the car by phantom motions (Trackmania Nations Forever, free version). **(D)** Patient using the Target Achievement Control (TAC) test as a rehabilitation tool.

Eight bipolar electrodes (self-adhesive Ag/AgCl, Ø = 1 cm, and ~2 cm inter-electrode distance) and the marker were placed around the stump, as shown in Figure 1. The location of the electrodes was defined by asking the patient to perform different movements and palpation of the corresponding muscular activity. We have empirically found that this procedure, rather than pre-defined selective placement, allows dealing with the difficulties of a commonly altered anatomy at the most distal part of the stump. The movements requested were hand open/close, wrist pro/supination, wrist flexion/extension and elbow flexion/extension.

The amplifiers used were developed in-house (MyoAmpF2F4-VGI8) with embedded active filtering: 4th order high-pass filter at 20 Hz; 2nd order low-pass filter at 400 Hz; and, Notch filter at 50 Hz. The signals were amplified with a gain of 2000 and digitalized at 2 kHz and 16 bits.

The protocol for myoelectric signals acquisition and processing is described in Ortiz-Catalan et al. (2013). The classifiers used were Linear Discriminant Analysis in a One-Vs-One topology (LDA-OVO), and Multi-layer Perceptron in a dedicated topology per degree of freedom (MLP-AAM), for individual and simultaneous movements, respectively. These classifiers have been shown to be successful at both tasks in real-time studies and are further described in Ortiz-Catalan et al. (in press).

### Intervention

Once the electrodes and marker were in place, and the quality of the EMG signals was verified by short real-time myoelectric recordings, the subject was asked to perform the eight movements while being guided on the length and timing of the contractions by a virtual limb. The instructions given to the patient were to perform the motions “as if he still had the missing limb,” thus aiming for physiologically appropriate myoelectric activity to be used for control. The LDA-OVO was trained with this information and the patient had a 10-min session in the AR environment in which the facilitator prompted the patient to perform the recorded movements one by one in random order (Figures 1A,B).

After the AR environment session, new EMG recordings were made for simultaneous movements using wrist pro/supination and elbow flexion/extension. This information was used to train the MLP-AAM, which real-time predictions were used to play a racing game (Trackmania Nations Forever, free version). The game was controlled by using wrist pro/supination to turn left/right, while elbow flexion/extension controlled the car acceleration/deceleration. After a gaming session of ~10 min, the same procedure was repeated for hand open/close and wrist flexion/extension, always in combination with elbow flexion/extension (Figure 1C). These combinations of motions were also used in the Target Achievement Control (TAC) test initially introduced by Simon et al. (2011b), with modifications described in Ortiz-Catalan et al. (in press). The artificial limb speed was two degrees/prediction (new predictions every 50 ms) and the target posture was displaced in 1 and 2 degrees of freedom (DoF). The velocity-ramp algorithm was used to facilitate controllability (Simon et al., 2011a). In this work, the TAC test was used for rehabilitation and training, rather than as an evaluation tool (Figure 1D).

Once the TAC tasks were completed, a new set of movements was recorded using all eight movements to conduct a “Motion Test” (Kuiken et al., 2009), as implemented in BioPatRec (Ortiz-Catalan et al., 2013). Similarly to the TAC test, the Motion Test was aimed as a rehabilitation tool. Questionnaires were administered by the facilitator at the end of the Motion Tests which concluded the session.

This protocol was applied once a week starting in March 2013 and this work includes the results up to week 18. In the last 5 weeks, two sessions a week were held, while in week eight no session was conducted because the patient was unavailable for reasons unrelated to treatment. A video showing examples of the interventions is available as Additional File 1.

## Results

**Figure 2:**
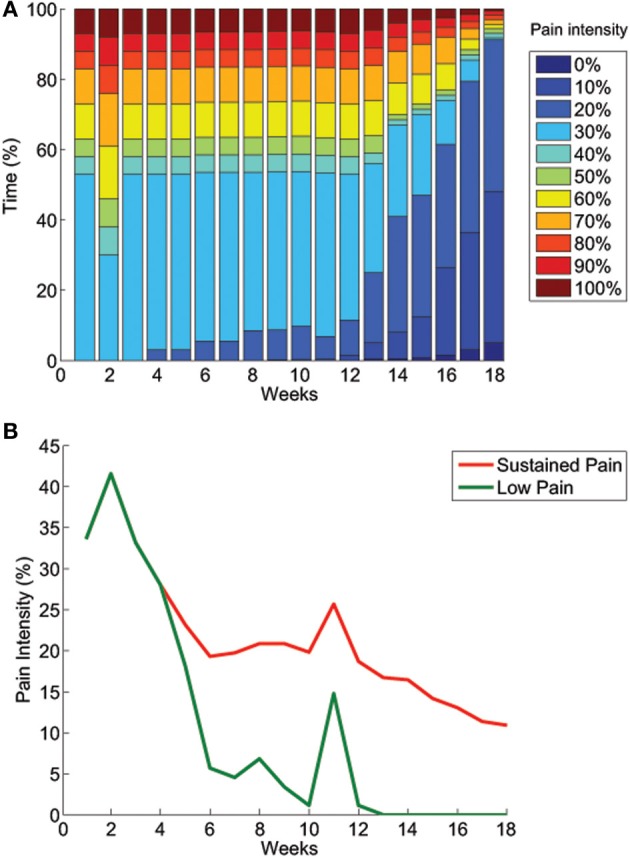
**Evolution of pain intensity over time. (A)** The distribution of pain intensity over time shows that at the beginning of the treatment, the patient had a sustained level of pain (~30%) during more than half of the time, and periods with higher levels of pain the rest of the time. Over the course of the treatment, a reduction of time at higher pain intensity levels was reported, as well as the appearance of periods of lower or absent pain. **(B)** The sustained level of pain was also the lowest pain perceived by the patient, and it gradually decreased to around 10% over the course of the interventions. Episodes of reduced pain started occurring after 4 weeks of treatment and gradually became pain-free periods. In week 11, a problem with his socket prosthesis caused him to use an old, tighter socket that had previously been shown to induce pain.

An increment in pain was reported by the patient after the first session, however, the pain decreased slightly below the original level in the second session, after which a slow yet consistent improvement was seen in the sustained level of pain. Figure 2 illustrates the progress in pain reduction. After 4 weeks, the patient reported starting to experience episodes of lower pain intensity. After 10 weeks, episodes of almost absent pain started occurring and this then developed into completely pain-free periods a couple of session later. This was reported by the patient as the most dramatic effect: “*These pain-free periods are something almost new to me and it is an extremely pleasant sensation*.” In addition, pain-free periods of 15–60 min were reported immediately after the rehabilitation sessions.

As the patient is very active in agricultural activities, despite his disabilities, he performs physical tasks that involve the use of his prosthesis. These activities often induced sessions of pain during the following days. Each week, the patient reported that the periods of pain that normally came in the days following the activities had been dramatically reduced and that he was able to work harder without being afflicted by PLP.

Surprisingly, the patient was capable of sequential control of three DoF from the first session, which evolved to four DoF and simultaneous control after four sessions. The patient reports that he is now able to control the motion of his phantom limb at will in the trained DoF. This is even possible in the absence of the visual feedback provided by the system, as is the case when he drives. More importantly, he reports being able to control (stop) the pain episodes considerably more effectively than before the interventions. Furthermore, he no longer wakes up at night due to PLP. The patient's life partner reports that it is her belief that “*My husband can live 10 years more than I expected, as pain now plays a less important role in his life and those close to him can see it*.”

We have previously observed that patients using BioPatRec reported a telescopic effect on the position of the phantom limb. The patient initially reported the perception of his phantom hand at the stump height, which over the course of the treatment extended to the anatomically unaltered position. Interestingly, when he rests his arm over a table producing sensory feedback, the perceived position of the hand moves back to the end of the stump. However, as soon as he starts producing phantom motions, the perceived position is once again restored to the unaltered anatomy. This is a phenomenon that is now permanently present and it indicates the complexity of self-perception and how it can be altered by sensory feedback and motor execution. It is worth noting that the presence of a phantom limb map on the stump is weak, mixed, and fairly difficult for the patient to identify, thus providing limited sensory information from the phantom hand.

The initial state of the phantom hand was described by the patient as a permanently, strongly closed fist and this has been the case for the last 48 years. After six sessions, this state evolved to a mid-open hand position, which coincides with the neutral (relaxed) position shown by the virtual hand. This is now the permanent perception of phantom hand posture and it is greatly appreciated by the patient (patient self-report). We do not have enough data to argue that the constant visualization of such position as the normal virtual state has influenced its perception as the default phantom limb state, or whether this is instead the result of the patient's skill at moving the phantom limb. In any case, the relaxation of such a stressed position occurred at the same time as the appearance of reduced pain periods and it could therefore be attributed as one of the causes of reduced PLP.

**Figure 3:**
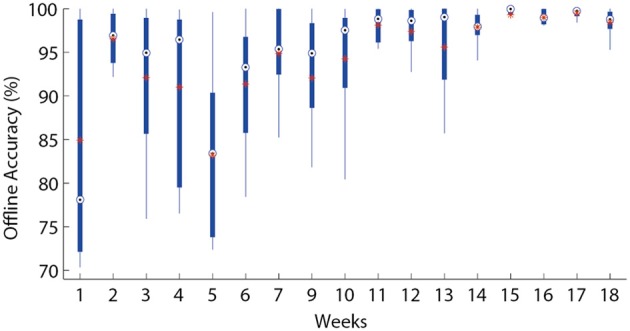
**Offline accuracy.** The offline discrimination accuracy over time is presented in box plots where the central mark represents the median value; the edges of the box are the 25th and 75th percentiles; the whiskers give the range of data values; “^*^” represent average values.

As expected, the ability of the patient to control the different motions improved over the course of the treatment. It is worth mentioning that no muscles directly responsible for the more distal movements were available due to the level of amputation (e.g., hand open and close). However, the patient was capable of voluntarily controlling the virtual limb to produce those motions. We hypothesize that the patterns of myoelectric activity produced by muscle synergies are distinctive enough to allow the classifiers to differentiate directly related movements from those occurring more distally at the hand. Figure 3 illustrates the learning curve through the improvement of the classification accuracy of nine classes (eight movements plus “no movement”). In this case, the classification accuracy indicates how well motions can be discerned from each other using information from the recorded sessions (offline), whereas the real-time performance once the patient has acquired experience with the system is shown in Table 1. Despite the relative low level of offline accuracy at the beginning of the treatment, the interventions were still possible because only a few movements were discriminated together for each rehabilitation task, thereby making the differentiation easier for the classifiers, i.e., only two DoF (four movements) were used for game control and the TAC test.

**Table 1:** **Motion test results**.

	**PLP patient**	**BioPatRec study**
# Movements	8	10
# Electrodes	8	4
Selection time (s)	0.56 (±0.14)	0.62 (±0.24)
Completion time (s)	1.71 (±0.15)	1.86 (±0.31)
Completion rate (%)	98.0 (±2)	87.4 (±11)
Real-time accuracy (%)	75 (±4.2)	67.1 (±10)

The motion test results after week 15 show performance comparable to that of 17 able-bodied subjects previously evaluated (Ortiz-Catalan et al., 2013), where the signal processing, features and motion test settings were the same. Although these results cannot be compared directly, they serve as an indication of the ability of the patient to produce distinctive motions in real-time. It is worthy of notice that the number of electrodes has been reduced to four since this report without a noticeable effect in classification performance.

## Discussion

A complete understanding of the root causes and underlying mechanisms of PLP has evaded the scientific community for decades (Nikolajsen and Jensen, 2001; Flor et al., 2006). This understanding will undoubtedly enable the creation of more effective treatments for the condition. In this context, the system proposed here provides empirical information on the effect of reactivating brain areas related to motor execution, enabling visual feedback that “tricks” the brain into believing that there is a limb responding to motor commands, and exercising the stump musculature, which is normally neglected. Mirror therapy is based on the assumption that visual feedback can potentially correct tactile deafferentation (to some degree) due to brain plasticity. Evidence has been reported on the correlation between cortical reorganization and PLP (Flor et al., 1995), which was further investigated to argue that extensive myoelectric prosthetic use prevents it, and thus reduces PLP (Lotze et al., 1999). The patient reports wearing his body-powered prosthesis all the time he is awake and he has done so for decades. In this case, although limited visual feedback is provided by the prosthesis, the prosthesis does not respond to physiologically appropriate commands and the motion of the missing limb is thus neglected. This might explain why, although the patient has used his prosthesis extensively, the PLP has remained. This underlines the importance of a congruent relationship between feedback and motor execution, as well as the intention to perform motion execution itself. All this is synergistically provided by our system.

In this case study, we present a system that can be used for PLP treatment and has had relative success in a patient with chronic PLP who had unsuccessfully explored several other treatments. Despite the fact that the pain has not disappeared completely at the time of this report, its reduction and temporal absence have considerably improved the patient's condition (patient self-report). It still remains to be seen whether the pain disappears completely after the long-term use of the system. The ideal medical treatment would be to administer it for a defined period of time and permanently cure the condition. We have intentionally avoided terminating the sessions to evaluate the long-term effect, as we feel this would be unethical, given the satisfaction reported by the patient after 48 years of chronic pain. As an alternative, the patient has been provided with a stand-alone system to be used at home and he has been instructed to use it at his own discretion. Follow-ups will be conducted every 2 months for a year and every year after that for 5 years.

The combination of myoelectric control of a virtual limb using physiologically appropriate signals, the enhanced illusion given by AR, and the entertainment provided by gaming has enabled the patient to develop the skill to control the motion of his phantom limb at will, even outside the lab. It is not clear whether this skill alone is enough to reduce PLP, because (1) this was acquired through visual feedback forcing the brain into the illusion that the limb is present, thus facilitating phantom limb motions (Brodie et al., 2003); and (2) the intervention inevitably results in motion intent and a workout of muscles at the stump which are normally neglected. It has been argued that the second factor alone is a cause of PLP relief (Sherman, 1980). The independent contribution of these two factors could be difficult to isolate in the proposed system. Motor intention alone has been shown to similarly reduce PLP when comparing mirror therapy with and without visual feedback (Brodie et al., 2007). When visual feedback was used, however, the capabilities of phantom limb motion increased. On the other hand, VR interventions where the controlling side is the amputated has shown signs of PLP relief, despite that the musculature at the stump was not directly involved (Cole et al., 2009). Treatment-wise, the combination presented here including all the latter was successful in a particular but complicated case, and it requires further investigation in a wider clinical study.

The possibility of decoding distal movements using muscles synergies was initially explored decades ago (Wirta et al., 1978). In 1982, Saridis and Gootee used pattern recognition to decode wrist pro/supination from biceps and triceps muscles (Saridis and Gootee, 1982), however, they reported that they were not able to decode hand open/close; possibly due to the limited number of electrodes used (2 bipolars). In our experience, patients can quickly learn to control a few distal motions and the results presented here suggest that they are able to develop that skill further to several motions. It is worth noting that one limitation of this non-invasive approach is that a certain degree of musculature is required, i.e., shoulder disarticulations would hardly be treatable unless they were recipients of targeted muscle reinnvervation (Kuiken et al., 2004, 2009). On the other hand, the proposed treatment can be used seamlessly in any patient requiring neuromuscular rehabilitation, in cases such as stroke and incomplete spinal cord injuries (Lee et al., 2011; Liu and Zhou, 2013), again, given the availability of myoelectric signals.

VR treatments are commonly justified and encouraged by the assumption that sensory stimulation boosts neuromuscular rehabilitation. At the current stage, the system employs only visual feedback stimuli, mostly due to the technical difficulties involved in providing proper somatosensory stimulation. In our experience, patients invariably prefer a virtual limb to any other visual feedback and we are therefore presently developing rehabilitation games based on AR that are specifically designed to exercise selected motions in a controlled manner.

The proposed system incorporates different advantages of computational rehabilitation systems, such as progress tracking, adjustable task difficulty, engaging rehabilitation tasks, and portability. Furthermore, the VR environment and all the source code necessary for motion prediction using sEMG (including game control) are freely available and open source in BioPatRec (Ortiz-Catalan et al., 2013), which aims to enable researchers worldwide to use this technology.

## Conclusions

PLP has historically being a difficult condition to treat and it affects the majority of amputees. In this work, we introduce a non-invasive technological proposal that combines the prediction of motion intent through the decoding of myoelectric signals, virtual and augmented reality, and gaming. As opposed to conventional mirror therapy, this system allows full range of motion and direct volitional control of the virtual limb, and it is applicable for bilateral amputees, in addition to having the known motivational benefits of gaming and progress tracking by computerized systems. This system is presented with a case study of a chronic PLP patient with known resistance to conventional PLP treatments. Having shown that the system has considerably increased the quality of life of a single patient, where other previous conventional treatments had proved unsuccessful, we believe that it offers sufficient justification to further explore its efficacy on a wider PLP population.

## Author contributions

Max Ortiz-Catalan designed the study, developed the motion prediction technology (software and hardware), performed the literature review, and drafted the manuscript. Nichlas Sander developed the virtual reality environment. Morten B. Kristoffersen developed the augmented reality environment. Max Ortiz-Catalan, Nichlas Sander, and Morten B. Kristoffersen performed the interventions and analyzed the results. Rickard Brånemark and Bo Håkansson supervised this research and revised the manuscript. All the authors have read and approved the final manuscript.

### Conflict of interest statement

In addition to governmental institutions, this work was partially funded by Integrum AB, which is currently investing in the advanced control of robotic prostheses. The technology for motion prediction was originally developed for prosthetic control and it is open source.

## Supplementary material

Additional File 1 **Video examples of the patient in the augmented reality environment, gaming, motion and TAC test**.
